# Thermal Decomposition, Low Temperature Phase Transitions and Vapor Pressure of Less Common Ionic Liquids Based on the Bis(trifuoromethanesulfonyl)imide Anion

**DOI:** 10.3390/ma15155255

**Published:** 2022-07-29

**Authors:** Annalisa Paolone, Boumediene Haddad, Didier Villemin, Mostefa Boumediene, Bekhaled Fetouhi, Mohammed Amin Assenine

**Affiliations:** 1 Consiglio Nazionale delle Ricerche, Istituto dei Sistemi Complessi, U.O.S. La Sapienza, Piazzale A. Moro 5, 00185 Roma, Italy; 2 Chemistry Laboratory of Synthesis, Properties, and Applications (CLSPA-Saida), University of Saida, Saida 20000, Algeria; haddadboumediene@yahoo.com (B.H.); m.boumediene68@gmail.com (M.B.); asseninema@gmail.com (M.A.A.); 3 Laboratoire de Chimie Moléculaire et Thio-Organique, École Nationale Supérieure D’ingénieurs de Caen (ENSICAEN), UMR 6507 CNRS, University of Caen, 6 bd Ml Juin, 14050 Caen, France; didier.villemin@ensicaen.fr; 4 Synthesis and Catalysis Laboratory (LSCT), Tiaret University, Tiaret 14000, Algeria; k.fetouhi@gmail.com; 5 Department of Chemistry, Université Djillali Liabes, BP89, Sidi-Bel-Abbes 22000, Algeria

**Keywords:** ionic liquids, decomposition temperature, phase transitions, vapor pressure

## Abstract

Four ionic liquids (ILs) based on the bis(trifluoromethanesulfonyl)imide (NTf_2_) anion were synthesized and characterized concerning their thermal stability, the occurrence of low temperature phase transitions and their volatility. All these physical quantities are highly important for possible applications. Both monocationic and dicationic ILs were considered. All ILs exhibit thermal stability exceeding 350 °C, an extremely high value, due to the presence of the NTf_2_ anion. Monocationic ILs can undergo crystallization, and they melt at 1 and 38 °C. On the contrary, dicationic ILs containing large positively charged ions display only a glass transition around −40 °C, without any crystallization or melting process; this fact is particularly important in view of the possibly low temperature applications of the dication ILs. The vapor pressure, p_v_, of the four ILs was measured by isothermal thermogravimetry in the temperature range between 250 and 325 °C; the lowest values of p_v_ were obtained for the two dicationic liquids, suggesting that they are particularly well suited for high temperature applications. The vaporization enthalpy was calculated through the Clausius–Clapeyron equation and was found in the range between ~140 and ~180 kJ/mol depending on the specific IL.

## 1. Introduction

Ionic liquids (ILs) are composed of ionic species, such as inorganic and/or organic anions and organic cations. By definition, their melting point is lower than 100 °C and in most cases they are liquid at room temperature or even below. They have attracted large interest due to envisaged applications in catalysis, solvation, electrochemistry, lubrication, synthesis and heat exchange. Among the many peculiar properties that make these materials so unique, one can include the large liquid temperature range of many ILs, their high thermal stability and low volatility. Decomposition temperatures and vapor pressures, p_v_, of ionic liquids are extremely important for applications; indeed high decomposition temperatures and low vapor pressures are essential for high temperature applications or applications in systems where a sudden temperature increase can be obtained as a consequence of the failure of a safety procedure.

For a long time, it was reported that ILs displayed a negligible or non measurable vapor pressure. In the last years, it has been clarified that most ILs have extremely low vapor pressures around room temperature, which can be measured by specialized set-up, such as Knudsen cells or quartz microbalance. For ILs having higher decomposition temperatures, other methods can be applied, such as isothermal thermogravimetry. The studies on vapor pressure of ILs started with the pioneering work of Paulechka et al. in 2005 that reported the vapor pressure of 1-butyl-3-methylimidazolium bis(trifluoromethanesulfonyl)imide (NTf_2_) measured by the Knudsen method in the temperature range between 180 and 245 °C [1]. Afterwards, a tendency to observe a decrease in the vapor pressure with an increase in the length of the alkyl chain of the imidazolium cation was reported [2,3,4]. The substitution of the NTf_2_ anion with PF_6_ was reported to lead to a decrease in the vapor pressure [5,6,7], while ILs with the dicyanamide anion showed higher values of p_v_ [8]. A large study of the vapor pressure of ILs conducted by means of isothermal thermogravimetry and a calibration with glycerol suggested that p_v_ has similar values for similar cation but decreases according to the presence of different anions in the following order Br (bromide) > DCA > BF_4_ > NTf_2_ > PF_6_ > TfO [9]. From vapour pressure measurements one can calculate the vaporization enthalpy and entropy [10,11,12,13,14,15], by means of the Clausius –Clapeyron equation:

(1) $$\ln\left(p\right)\propto -\frac{{∆}_{l}^{g}H_{m}^{{}^{\circ}}}{\mathrm{RT}}+\frac{{∆}_{l}^{g}S_{m}^{{}^{\circ}}}{R}$$

where R is the gas constant and ${∆}_{l}^{g}H_{m}^{{}^{\circ}}$ and ${∆}_{l}^{g}S_{m}^{{}^{\circ}}$ are the standard molar vaporization enthalpy and entropy. In many cases, corrections to the obtained values were applied in order to refer the standard vaporization entropy and enthalpy values to room temperature (25 °C) [10,11,12,13,14,15].

In the last two years, a large number of measurements of vapor pressure of ILs and of derived vaporization enthalpy and entropy values was reported, mainly based on experiments conducted just above room temperature by means of the quartz microbalance technique. These experiments extended the previous measurements to less exploited ions, such as tetraalkylphosphonium cations, which render ILs significantly more volatile than alkyl-imidazolium ILs with comparable chain length [16]. On the contrary, ILs with the NTf_2_ anion and tetrahydrothiophene based cations were reported to have p_v_ similar to the corresponding series with imidazolium cations [17]. The cations [Ph4P] and Cs decrease the vapor pressure with respect to the imidazolium equivalents [18]. Recently the vapor pressure of ILs based on amino acids, namely [C_4_Dmim]Gly and [C_4_Dmim]Ala, was also measured [19].

Regarding the influence of the anions on p_v_, it was reported that with increasing fluorination of the anion, passing from the NTf_2_ to the BETI anion, the volatility increases [20]. Other studies pointed out that the absolute vapor pressures of ILs containing the tris(pentafluoroethyl)trifluorophosphate anion or the nonafluorobutane-1-sulfonate anions do not significantly differ from those of the liquids containing the NTf_2_ anion [21,22]. The fluorinated FSI anion was reported to significantly decrease [23] or increase [24] the vapor pressure compared to NTf_2_. ILs containing the diethyl phosphate [25], thiocyanate [26], methanesulfonate [27], formate and acetate [28] anions displayed high values of the vapor pressure, while those based on the iodide, bromide or chlorine anions have extremely low values of p_v_ [29,30]. ILs containing the B(CN)_4_ anion have p_v_ comparable to that of NTf_2_ samples [31]. Bulow et al. reported a comparison of the vapor pressure of ILs containing the same 1-ethyl-3-methyl-imidazolium cation and a series of anions, such as PF_6_, B(CN)_4_, (C_2_H_5_O)_2_PO_2_, NTf_2_, SCN, CF_3_CO_2_, CF_3_SO_3_, BF_4_, C(CN)_3_, (C_2_F_5_)_3_PF_3_, 4-CH_3_-Ph-SO_3_ [32]. Finally, in the last couple of years some models were also proposed to derive the vapor pressure and vaporization enthalpy values from the knowledge of the structure of ILs [33,34,35].

In the present paper, we will focus on four ILs based on the NTf_2_ anion, whose vapor pressure was not previously reported. The choice was motivated by two reasons: on the one hand, ILs based on the NTf_2_ anion are known to possess high thermal stability; on the other hand, we wanted to extend vapor pressure measurements to dicationic ILs, which are known to possess higher melting points and thermal stability compared to their monocationic counterparts.

## 2. Materials and Methods

### 2.1. Synthesis of the Samples

The reagents used in this study were: 1,4-diazabicyclo [2.2.2] octane, pyridine, 1-methyl-1H-imidazole (>99%), 1-butyl-1H-imidazole (>99%), bromodecane (98%), α,α′-dichloro-m-xylene (98%), hexyl bromide (98%), α,α′-dibromo-p-xylene (98%), lithium bis(trifluoromethylsulfonyl)imide (99%), ethylacetate, diethylether, and N,N-dimethylformamide. They were purchased from Fluka (Merck KGaA, Darmstadt, Germany) and used as received. Deionized H_2_O was obtained with a Millipore ion-exchange resin deionizer (Merck KGaA, Darmstadt, Germany).

#### 2.1.1. Synthesis of Halogenated ILs

The ionic liquids based on 1-decyl-1,4-diazabicyclo [2.2.2] octan-1-ium [DABCO10^+^], 3,3′-dimethyl-1,1′-(1,3-phenylenedimethylene)-bis(1H-imidazolium) [m-C_6_H_4_(CH_2_ImMe)^+2^], 3,3’-dibutyl-1,1’-(1,4-phenylenedimethylene)-bis(1H-imidazolium) [p-C_6_H_4_ (CH_2_ImBu)^+2^] and 1-hexylpyridinium [C_6_Py^+^] with chloride or bromide anions were prepared through a procedure described in detail elsewhere [36,37,38,39] and reported in a shorter form in the present paper. The syntheses are based on an alkylation reaction of precursors and alkyl halides.

#### 2.1.2. Synthesis of 1-Decyl-1,4-diazabicyclo [2.2.2] Octan-1-ium Bromide [DABCO10^+^][Br^−^]

A mixture of 1,4-diazabicyclo [2.2.2] octane (DABCO, 10 g, 89.1 mmol) and 1-bromodecane (19.70 g, 90.1 mmol) were dissolved in AcOEt (125 mL) before being stirred at room temperature (25 °C) for 24 h. The obtained [DABCO10^+^][Br^−^] was evaporated under vacuum to remove AcOEt and washed then with diethyl ether (100 mL) to give the 1-decyl-1,4-diazabicyclo [2.2.2] octan-1-ium bromide [DABCO10^+^][Br^−^] as a yellowish solid (27.05 g). The yield of this reaction was 57%.

#### 2.1.3. Synthesis of 3,3′-Dimethyl-1,1′-(1,3-phenylenedimethylene)-bis(1H-imidazolium) Dichloride [m-C_6_H_4_(CH_2_ImMe)^+2^][Cl^−^]_2_

IL named [m-C_6_H_4_(CH_2_ImMe)^+2^][Cl^−^]_2_ was prepared under microwave irradiation, from the mixture: 4.26 g (4.13 mL, 50 mmol) of 1-methylimidazole and 2.27 g (10 mmol) of the m-xylene dichloride in N,N-dimethylformamide (3 mL) at 100 °C for 3 min. The resulting mixture was washed three times upon addition of diethyl ether (100 mL), and then dried in vacuo (<1 mbar) for 8 h, to give a white hygroscopic solid compound in high yield (≈96%). The choice of using microwave irradiation was motivated by the faster reaction time and higher yield obtained by this technique.

#### 2.1.4. Synthesis of 3,3’-Dibutyl-1,1’-(1,4-phenylenedimethylene)-bis (1H-imidazolium) Dibromide [p-C_6_H_4_ (CH_2_ImBu)^+2^][Br^−^]_2_

A mixture of 1 p-xylene dibromide (2.64 g, 10 mmol) and 1-butylimidazole (2.48 g, 20 mmol) was heated at 150 °C for 24 h in the presence of 3 mL of N,N-dimethylformamide (DMF). After cooling at room temperature, the resulting mixture was washed with diethylether (3 × 10 mL) to remove the unreacted starting reagents, followed by evaporation under reduced vacuum to eliminate any possible volatile impurities; we come to obtain the product [p-C_6_H_4_(CH_2_ImBu)][Br]_2_ in the form of a white hygroscopic solid.

#### 2.1.5. Synthesis of 1-Hexylpyridinium Bromide [C_6_Py^+^][Br^−^] IL

The chemical reaction of pyridine (16.16 mL, 2 × 10^−1^ M) with hexyl bromide (28.07 mL, 2 × 10^−1^ M) in the presence of 50 mL of toluene yields transfer of the hexyl group to the pyridinium ring and results in pyridinium bromide [C_6_Py^+^][Br^−^] as a colorless solid. The yield of the reaction was 85%.

### 2.2. Synthesis of Fluorinated ILs

Following procedures described in the literature [40,41], the four halogenated ILs were subjected to anion exchange from halide to bis(trifluoromethanesulfonyl)imide. The reaction of lithium bis(trifluoromethanesulfonyl)imide with [DABCO10^+^][Br^−^], [m-C_6_H_4_(CH_2_ImMe)^+2^][Cl^−^]_2_, [p-C_6_H_4_(CH_2_ImBu)^+2^][Br^−^]_2_ and [C_6_Py^+^][Br^−^] in water leads to the corresponding ionic liquids [DABCO10^+^][NTf_2_^−^], [m-C_6_H_4_(CH_2_ImMe)^+2^][NTf_2_^−^]_2_, [p-C_6_H_4_ (CH_2_ImBu)^+2^][NTf_2_^−^]_2_ and [C_6_Py^+^][NTf_2_^−^]. Table 1 summarizes the names, acronyms and structures of the investigated cations. To avoid possible water contamination, the four ILs were dried in a high-vacuum line (*p* < 1 × 10^−5^ bar) and on phosphorus pentoxide for 4 days at ~40 °C. The structures of the obtained ILs were confirmed using ^1^H, ^13^C, ^19^F-NMR and FT-IR spectroscopy. The data also confirmed the absence of significant impurities, e.g., residuals of the reactants or by-products. The spectroscopic data are given below.

### 2.3. NMR Analysis

The NMR data, including ^1^H-NMR (500 MHz), ^13^C-NMR (125.75 MHz), and ^19^F-NMR (470.62 MHz) spectra, were recorded by using a Bruker DRX 500 MHz spectrometer. The chemical shifts (δ) are given in ppm and referenced to the internal solvent signal, namely TMS, CDCl_3_, DMSO-d_6_, CD_3_OD and CFCl_3_, respectively. The coupling constants (J) are expressed in Hertz (Hz). As examples, the NMR spectra of [DABCO10^+^][NTf_2_^−^], [m-C_6_H_4_(CH_2_ImMe)^+2^][NTf_2_^−^]_2_ are reported in the Supplementing Information as Figures S1 and S2.

#### 2.3.1. [DABCO10^+^][NTf_2_^−^]

**^1^H–NMR** (CDCl_3_) δ_H_ (ppm) = 3.32–3.29 (t, ^3^J = 7.4 Hz, 6H), 3.22–3.20 (t, ^3^J = 8 Hz, 2H), 3.16 (t, ^3^J = 7.8 Hz, 6H), 1.75–1.72 (m, 2H), 1.70–1.69 (m, 2H), 1.31–1.29 (m, 2H), 1.26 (m, 10H), 0.89–0.86 (t, ^3^J = 7.5 Hz, 3H). **^13^C–NMR** (CDCl_3_) δ_C_ (ppm) = 65.05, 52.47, 45.13, 31.70, 29.23, 28.92, 26.02, 22.69, 21.78, 14.02. **^19^F–NMR** (DMSO-d_6_) δ_F_ (ppm) = −78.91 (s, [NT**f**_2_^−^]).

#### 2.3.2. [m-C_6_H_4_(CH_2_ImMe)^2+^][NTf_2_^−^]_2_

**^1^H–NMR** (DMSO-d_6_) δ_H_ (ppm) = 9.12 (s, 2H, NCHN), 7.69–7.67 (d, 4H, NCHCHN), 7.40–7.49 (m, 4H, C_6_H_4_), 5.41 (s, 4H, –CH_2_–), 3.85 (s, 6H, 2 × CH_3_). **^13^C–NMR** (DMSO-d_6_) δ_C_ (ppm) = 36.15, 51.96, 122.71, 124.46, 128.44, 129.08, 130.29, 135.86, 137.08. **^19^F–NMR** (DMSO-d_6_) δ_F_ (ppm) = −79.36 (s, [NT**f**_2_^−^]).

#### 2.3.3. [p-C_6_H_4_(CH_2_ImBu)^+2^][NTf_2_^−^]_2_

**^1^H–NMR** (DMSO–d_6_) δ_H_ (ppm) = ^1^H–NMR (DMSO–d_6_) δ_H_ (ppm): 1.02 (t, J = 7.2 Hz, 3H,CH_3_); 1.43 (m, 2H, CH_2_); 1.91 (m, 2H, CH_2_); 4.25 (t, J = 7.5 Hz, 2H, CH_2_N); 5.51 (s, 4H, CH_2Ar_), 7.56 (d, 4H, CH_Im_), 7.76 (d, 4H, CH_Im_), 7.89 (s, 2H, CH_Ar_), 7.95 (s, 2H, CH_Ar_), 9.51 (s,2H, NCHN). **^13^C–NMR** (DMSO–d_6_) δ_C_ (ppm) = 13.17 (CH_3_); 19.31 (CH_2_); 31.87 (CH_2_); 50.06 (CH_2_N); 52.12 (CH_2Ar_); 122.87 (CH_Im_), 124.61 (CH_Im_), 127.87, 137.38, 140.18 (NCHCH), 143.2 (CF_3_). **^19^F–NMR** (DMSO–d_6_) δ_F_ (ppm) = −78.75 (s, [NT**f**_2_^−^]).

#### 2.3.4. [C_6_Py^+^][NTf_2_^−^]

**^1^H–NMR** (CD_3_OD) δ_H_ (ppm) = 8.82 (d, 2H, Pyr_2,6_), 8.47 (t, 1H, Pyr_4_), 8.97 (d/d, 2H, Pyr_3,5_), 4.52 (t, 2H, N-CH_2_), 1.94–1.86 (m, 8H, 4CH_2_), 0.87 (t, 3H, CH_3_). **^13^C–NMR** (CD_3_OD) δ_C_ (ppm) = 146.64 (Py_4_), 145.56 (Py_2,6_), 129.35 (Py_3,5_), 62.93 (CH_2_-N), 32.11 (CH_2_), 31.87 (CH_2_), 26.80 (CH_2_), 23.12 (CH_2_), 13.90 (CH_3_). **^19^F–NMR** (CD_3_OD) δ_F_ (ppm) = −78.91 ppm (s, [NT**f**_2_^−^]).

The IR spectra of the four ionic liquids are reported in the Supplementing Information, as Figure S3. The main absorption bands were attributed in references [37] and [38]. Most of them correspond to the movements of the [NTf_2_] anion.

### 2.4. Thermal Characterization of the Samples

Thermogravimetry measurements were performed by means of a Setaram Setsys Evolution 1200 TGA system (KEP Technologies Group, Mougins—Sophia Antipolis, France) between room temperature and 800 °C, in an inert helium flux of 60 mL/min. Either experiments in scanning mode at 10 °C/min or in isothermal mode were conducted. The initial mass of the samples for the two types of experiments was in the range of 15 mg and 100 mg, respectively.

The vapor pressure of the liquids was obtained from measurements of the mass variation as a function of time in isothermal mode at selected temperatures between 250 and 325 °C, well below the decomposition temperature measured in scanning mode, as described in [24]. Briefly, the slope, *k*, of the variation of the mass on time was determined by means of a linear fit for each isothermal measurement. As suggested by the previous literature [9,24], the vapour pressure, p_v_, was calculated as:

ln p_v_(bar) = *a* ln *k* + *b* (2)

where *a* and *b* are coefficients determined by the calibration of the TGA apparatus with glycerol [24]. For our instrument, as reported in one of our previous papers, *a* = 1.05 ± 0.02 and *b* = −3.77 ± 0.05 [24].

Differential scanning calorimetry measurements were conducted by means of a Mettler Toledo DSC 3 system (Mettler Toledo, Columbus, OH, USA) equipped with a Huber TC100 Cryocooler (Peter Huber Kältemaschinenbau AG, Offenburg, Germany). An argon flux of 50 mL/min and a temperature rate of 5 °C/min were applied. An initial mass of the order of 10 mg was used for each experiment.

Before experiments, the samples were dried to avoid water contamination; for thermogravimetry measurements the samples were kept at 100 °C in the TGA apparatus in argon atmosphere, and then cooled to room temperature before experiments started, avoiding air exposure. For small quantities of samples 30 min at 100 °C were sufficient to remove water (no mass variation occurred after that time), while for the larger samples used for isothermal measurements, longer treatments were necessary (4 or 5 h). For DSC measurements, the small mass of sample was heated in the DSC for 1 h at 100 °C in argon and no subsequent air exposure was allowed. The glass transition and melting temperatures of the ionic liquids were defined as the position of the minimum of the respective endothermic peaks.

## 3. Results and Discussion

The experimental results obtained in the present work are reported in the following sub-sections divided according to the measured physical quantity: the decomposition temperature, which defines the maximum temperature at which the ILs can be used, is shown in Section 3.1; the occurrence of low temperature phase transitions, which define the minimum allowed temperature for applications, is shown in Section 3.2; the vapor pressure, which shows the volatility of the ILs, is shown in Section 3.3.

### 3.1. Thermal Decomposition

**Figure 1:**
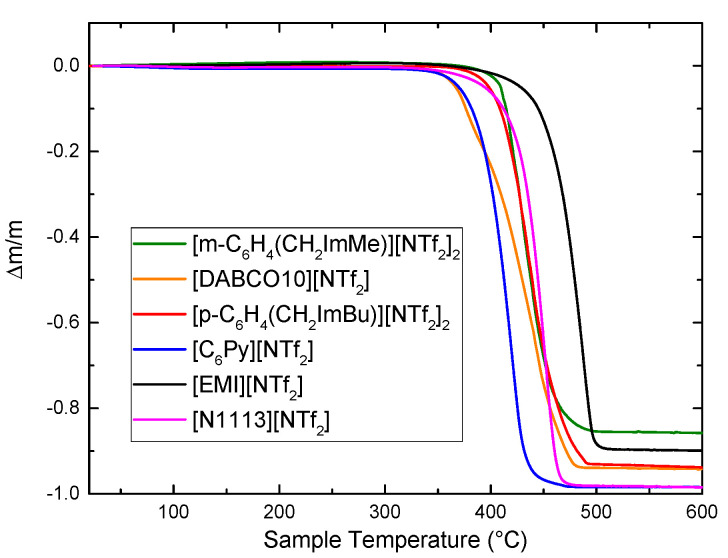
TGA curves of the four ILs investigated in this work; for comparison the TGA curves of [EMI][NTf_2_] and [N1113][NTf_2_] are reported [24].

The TGA curves of the investigated ionic liquids are reported in Figure 1, together with the curves of more largely investigated 1-ethyl-3-methylimidazolium bis(trifluoromethanesulfonyl)imide ([EMI][NTf_2_]) and N-trimethyl-N-propylammonium bis(trifluoromethanesulfonyl)imide ([N1113][NTf_2_]) [24], for comparison purposes.

All the ILs are highly stable, as a large mass loss is visible only above at least 350 °C. Table 2 reports the decomposition temperature, T_d_, here defined as the temperature at which a mass loss of 2% is observed. It can be noted that T_d_ increases passing from 353 °C of [C_6_Py][NTf_2_], to 354 °C of [DABCO10][NTf_2_], 384 °C of [p-C_6_H_4_(CH_2_ImBu)^+2^][NTf_2_^−^]_2_ and 398 °C for [m-C_6_H_4_(CH_2_ImMe)^2+^][NTf_2_^−^]_2_. This last temperature is only slightly lower than the decomposition temperature of [EMI][NTf_2_] (404 °C), which is known for its high thermal stability [42,43]. The decomposition of all liquids apparently occurs in a single step, which is completed below 500 °C. The extremely high decomposition temperatures, found in the investigated ionic liquids, is a consequence of the presence of the NTf_2_ anion, which usually provides higher thermal stability compared to other anions, such as BF_4_ or halides [43].

The TGA curve of [C_6_Py][NTf_2_] is quite similar to that previously reported in [44], even though a slightly different temperature rate was used by those authors (15 °C/min). Bitter et al. reported that an increase in the length of the pyridinium alkyl chain induced a higher thermal stability [44].

DABCO-based ionic liquids with alkyl chains longer than the one here investigated, i.e., with at least 12 carbon atoms, were recently synthesized for electrochemical applications [45]. The thermal stability of all ionic liquids with 12 to 20 carbon atoms in the alkyl chain was similar and a 1% mass loss was observed around 340 °C [45], a value in close agreement with that here reported for [DABCO10][NTf_2_]. [DABCO8][PF_6_], instead, displayed a reduced thermal stability, as it started to decompose around 271 °C [46].

Concerning m-xylene based ILs, it was previously evidenced that the anion has a large influence on the decomposition temperature: it increases from 210 °C for BF_4_, to 240 °C for Cl, 354 °C for PF_6_ and 398 °C for NTf_2_ [37].

**Table 2:** Decomposition (T_d_), glass transition (T_g_), melting (T_m_) temperatures and vaporization enthalpy (${∆}_{l}^{g}H_{m}^{{}^{\circ}}$ ) of the investigated samples.

Ionic Liquid	T_d_ (°C)	T_g_ (°C)	T_m_ (°C)	${∆}_{l}^{g}H_{m}^{{}^{\circ}}(\mathrm{kJ}/\mathrm{mol})$
[EMI][NTf_2_]	404 [24]		−14 [47]	
[m- C_6_H_4_(CH_2_ImMe)][NTf_2_]_2_	398 [37]	−40		136 ± 18
[p-C_6_H_4_(CH_2_ImBu)][NTf_2_]_2_	384	−42		147 ± 13
[N1113][NTf_2_]	367 [24]		13 [48]	
[DABCO10][NTf_2_]	354		38	147 ± 12
[C_6_Py][NTf_2_]	353	−75	1	180 ± 17

### 3.2. Low Temperature Phase Transitions

**Figure 2:**
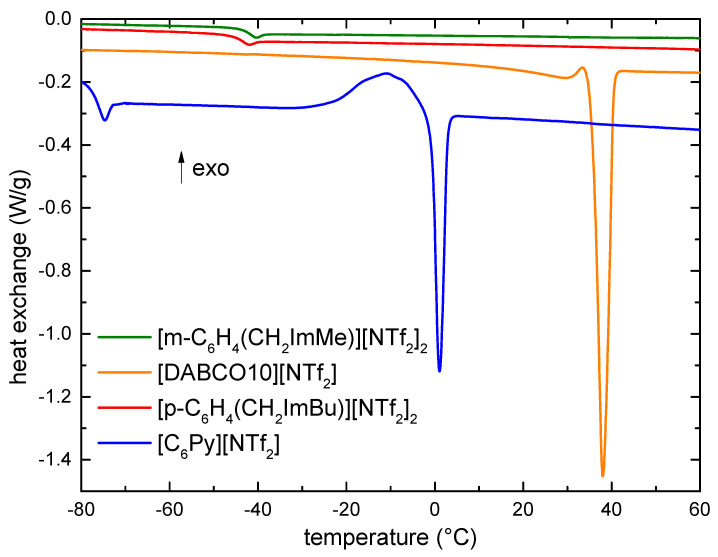
DSC traces measured on heating of the investigated ionic liquids.

Three of the four ILs are liquid at room temperature, while the fourth ([DABCO10][NTf_2_]) melts slightly above. Therefore, it is interesting to investigate the liquid range of these ILs in view of the possible applications. Figure 2 shows the DSC traces measured on heating for the four ILs.

[DABCO10][NTf_2_] crystallizes on cooling, starting around 20 °C. No further phase transitions are observable at lower temperatures. On heating, [DABCO10][NTf_2_] displays a single melting process, with the negative maximum of the heat exchange located at 38 °C. The melting point of [DABCO][NTf_2_] ILs with longer alkyl chains was reported in [45]. These authors suggested the occurrence of two melting transitions except for the shortest compound (*n* = 12). An increase in T_m_ was observed passing from ~37 to ~50 °C while the length of the chain increased from *n*= 12 to 20 [45]. The melting temperature here obtained for [DABCO10][NTf_2_] is in line with the values previously reported for samples with longer alkyl chains.

[C_6_Py][NTf_2_] shows a more complex behavior, as it does not show crystallization on cooling, but it undergoes a glass transition as witnessed by the endothermic peak measured on heating around −75 °C (see Figure 2). On further heating, at higher temperatures, a clear cold crystallization starts around −30 °C; around −5 °C, an endothermic process begins, corresponding to the melting of the sample, and the negative maximum of the heat exchange occurs at 1 °C. These values are in agreement with a previous piece of work [49].

The two dicationic ionic liquids, [p-C_6_H_4_(CH_2_ImBu)][NTf_2_]_2_ and [m-C_6_H_4_(CH_2_ImMe)][NTf_2_]_2_, display only a glass transition around −42 and −40 °C, respectively; no indication of crystallization (on cooling) or melting (on heating) can be found in their DSC curves. The absence of a crystallization was corroborated by a DSC measurement of [m-C_6_H_4_(CH_2_ImMe)][NTf_2_]_2_ conducted with a much lower scanning rate (1 °C/min), which excluded a kinetic hindrance of the crystallization. Moreover, when measured with the lower scanning rate, [m-C_6_H_4_(CH_2_ImMe)][NTf_2_]_2_ showed only the glass transition around −43 °C (see Figure S4 of the Supplementing Information). The fact that dicationic ionic liquids do not crystallize is particularly important in view of low temperatures, for which the occurrence of a liquid state is necessary.

It is particularly interesting to note the absence of crystallization in [m-C_6_H_4_(CH_2_ImMe)][NTf_2_]_2_, as its analogues containing smaller anions are solid up to high temperatures; indeed, the melting point of samples with the same cation increases from 136 °C for the BF_4_ anion, to 150 °C for PF_6_ and 190 °C for Cl. A possible explanation for the absence of the crystallization in both [p-C_6_H_4_(CH_2_ImBu)][NTf_2_]_2_ and [m-C_6_H_4_(CH_2_ImMe)][NTf_2_]_2_ could be found in the large steric hindrance of the two large ions composing each of them. The steric hinderance of crystallization of ionic liquids was already reported, for example, in the case of different per(fluoroalkylsulfonyl)imide anions, having symmetric, moderately asymmetric, highly asymmetric or cyclic structures [50]. The ILs possessing highly asymmetric anions could not crystallize, while those having more symmetric anions (in that case also NTf_2_) could undergo a crystallization process [50]. In the present case, we cannot ascribe the steric hinderance only to the anion because most ILs containing NTf_2_ crystallize at low temperatures but are more likely to respond tothe presence of the large cations and the difference in dimensions between anion and cation, which prevents a good packing of the ions in a crystal structure.

### 3.3. Vapor Pressure and Vaporization Enthalpy

**Figure 3:**
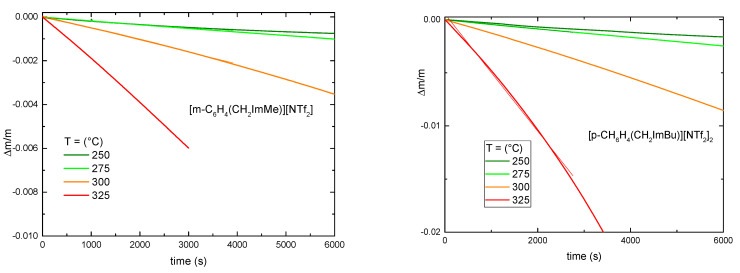

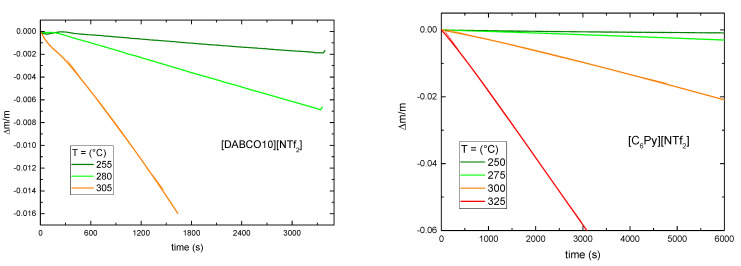
Time dependence of the relative mass loss at selected temperatures for the four ionic liquids (thicker lines) and best fit lines (thinner lines).

In order to measure the vapor pressure of the four ionic liquids, isothermal measurements were performed between 175 and 325 °C in steps of 25 °C, well below the decomposition temperature of the ILs reported in Table 2. Figure 3 reports all the measurements where an appreciable mass variation is observed; practically, all ILs started to exhibit a measurable mass loss on long times around 250 °C.

**Figure 4:**
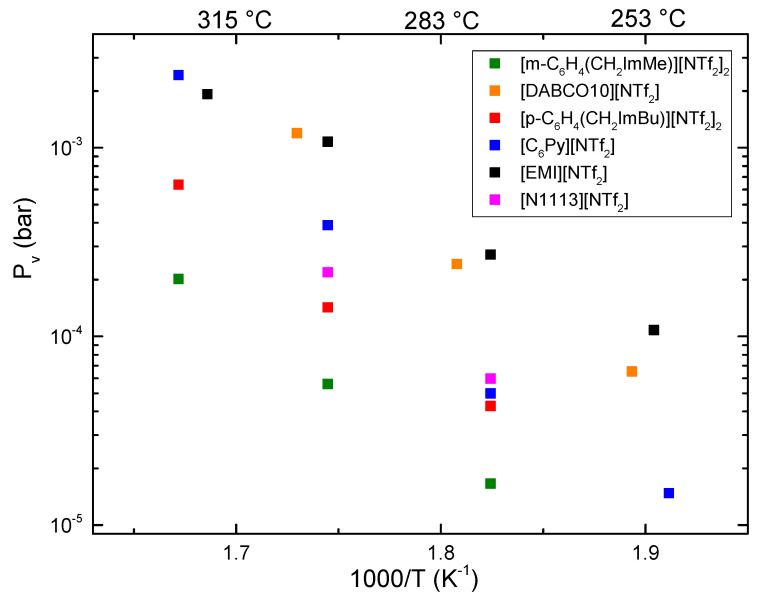
Vapor pressure of the four investigated liquids, as a function of the inverse of absolute temperature. For comparison the values obtained for [EMI][NTf_2_] and [N1113][NTf_2_] are also reported [24].

A fit of the data of Figure 3 allowed us to calculate the slope, *k*, of the linear relationship between the relative mass variation, Δm/m, and the time, t. Using Equation (2) the vapor pressure values were calculated. They are reported in Figure 4. For comparison, in Figure 4 the vapor pressure of [EMI][NTf_2_] and [N1113][NTf_2_] were also reported [24].

The ionic liquids containing the NTf_2_ anion generally have low vapor pressures [2]; ILs containing BF_4_ and PF_6_ anions display slightly lower p_v_ [9], while ILs with other anions generally display much higher values [8,9]. However, ILs containing BF_4_ anions usually have decomposition temperatures lower than those with PF_6_ or NTf_2_ anion [9]. Longer alkyl chains of the cations were shown to decrease the vapor pressure values, both in imidazolium- and ammonium-based ILs [3,4,9]. In our previous work [24], we showed that among NTf_2_-based ILs, those with an ammonium cation had p_v_ values lower than those based on the EMI cation, and [N1113][NTf_2_] displayed the lowest values among the investigated ILs [24].

Figure 4 shows that [DABCO10][NTf_2_] has vapor pressures slightly lower than that of [EMI][NTf_2_] and higher than [N1113][NTf_2_]. [C_6_Py][NTf_2_] has p_v_ close to that of [N1113][NTf_2_] at 275 °C and a higher value at 300 °C. On the contrary, both dicationic ILs, ([p-C_6_H_4_(CH_2_ImBu)][NTf_2_]_2_ and [m-C_6_H_4_(CH_2_ImMe)][NTf_2_]_2_), exhibit vapor pressure values lower than those of [N1113][NTf_2_]. In particular, [m-C_6_H_4_(CH_2_ImMe)][NTf_2_]_2_ has the lowest vapor pressure values in the whole temperature range. The vapor pressure of another dicationic ionic liquid was previously reported: [C_3_(C_1_Im)_2_][NTf_2_] has p_v_ = 4 × 10^−6^ bar at 300 °C and 4.7 × 10^−5^ bar at 332 °C [9]. It is important to stress that in the present study, the vapor pressure of both dication ionic liquids is lower than that of the other ILs. This fact displays that dicationic ILs not only posses high decomposition temperature, but also quite low vaporization rates, which is important for possible high temperature applications of this class of ILs. It is important to stress that the high decomposition temperatures and the low vapor pressure values at such high temperatures are due to the presence in all the investigated ILs of the highly stable NTf_2_ anion.

Finally, from a linear fit of the dependence of the logarithm of the vapor pressure from the inverse of the absolute temperature, one can obtain, by means of the Clausius–Clapeyron equation (Equation (1)), the value of the mean vaporization enthalpy in the considered temperature range (see Table 2). For the presently investigated ionic liquids, it varies from 136 ± 18 kJ/mol for m-C_6_H_4_(CH_2_ImMe)][NTf_2_]_2_ to 147 ± 12 and 147 ± 13 kJ/mol for [DABCO10][NTf_2_] and [p-C_6_H_4_(CH_2_ImBu)][NTf_2_]_2_, respectively, and finally to 180 ± 17 kJ/mol for [C_6_Py][NTf_2_]. These values are consistent with those previously reported for the other ionic liquids [16,17,18,19,20,21,22,23,24,25,26,27,28,29,30,31,32,33,34,35].

## 4. Conclusions

In this work, we synthesized four ionic liquids containing the NTf_2_ anion. The thermal stability of all these ILs is extremely high, as it exceeds 350 °C. [DABCO10][NTf_2_] and [C_6_Py][NTf_2_] display a melting temperature of 38 and 1 °C, respectively, while the two dicationic ILs, [m-C_6_H_4_(CH_2_ImMe)][NTf_2_]_2_ and [p-C_6_H_4_(CH_2_ImBu)][NTf_2_], show a glass transition around −40 °C and no sign of crystallization or melting. The absence of crystallization in dicationic ILs is particularly important in view of possible applications, as their liquid range is extremely large. The vapor pressures of the four ILs were measured between 250 and 325 °C, well below the decomposition temperature. The lowest values of p_v_ are obtained for the two dicationic liquids, while the vapor pressure of the monocationic ILs is comparable to that of the more extensively investigated [EMI][NTf_2_] and [N1113][NTf_2_] liquids. The low p_v_ values for the dicationic ILs is well suited for high temperature applications. The vaporization enthalpy was obtained through the Clausius–Clapeyron equation: an increase from 136 to 147 and 180 kJ/mol was observed passing from [m-C_6_H_4_(CH_2_ImMe)][NTf_2_]_2_ to [DABCO10][NTf_2_] and [p-C_6_H_4_(CH_2_ImBu)][NTf_2_]_2_ and finally to [C_6_Py][NTf_2_].

## Figures and Tables

**Table 1:** Name, acronym and structure of the different cations of the four ionic liquids.

Name of Cation and Acronym	Structure of Cation
[DABCO_10_^+^]:1-decyl-1,4-diazabicyclo[2.2.2]octan-1-ium	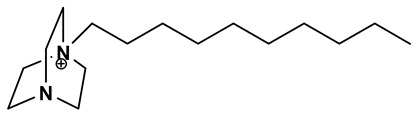
[m-C_6_H_4_(CH_2_ImMe)^+2^]:3,3′-dimethyl-1,1′-(1,3-phenylenedimethylene)-bis(1H-imidazolium)	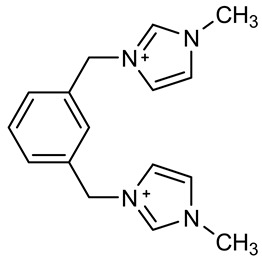
[p-C_6_H_4_(CH_2_ImBu)_2_]^+^^2^:3,3’-dibutyl-1,1’-(1,4-phenylenedimethylene)-bis (1H-imidazolium)	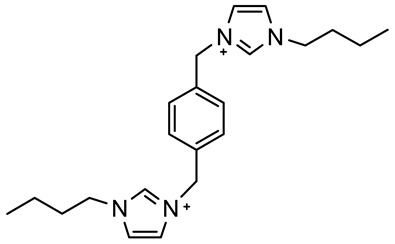
[C_6_Py^+^]:1-hexylpyridinium	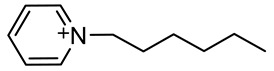

## Data Availability

Data are contained within the article or Supplementary Materials.

## Supplementary Materials

The following supporting information can be downloaded at: https://www.mdpi.com/article/10.3390/ma15155255/s1, Figure S1. ^1^H NMR (500 MHz) (a), ^13^C NMR (125.75 MHz) (b) and ^19^F NMR (470.62 MHz) (c) of [m-C_6_H_4_(CH_2_ImMe)^2+^][NTf_2_^−^]_2_; Figure S2. ^1^H NMR (500 MHz) (a), ^13^C NMR (125.75 MHz) (b) and ^19^F NMR (470.62 MHz) (c) of [DABCO10^+^][NTf_2_^−^]; Figure S3. Infrared absorption spectra of the four ionic liquids; Figure S4. DSC trace of [m-C_6_H_4_(CH_2_ImMe)^2+^][NTf_2_^−^]_2_ measured with a scanning rate of 1 °C/min.
