# Supplementary material for: Thermal Decomposition, Low Temperature Phase Transitions and Vapor Pressure of Less Common Ionic Liquids Based on the Bis(trifuoromethanesulfonyl)imide Anion
Source: Materials (Basel). 2022 Jul 29;15(15):5255. doi: 10.3390/ma15155255 (PMC9370012; doi:10.3390/ma15155255)
Supplement: Supplementary file 1 [file materials-15-05255-s001.zip › materials-1841213-supplementary.pdf]

materials

MDPI

Supplementing Information

Thermal decomposition, low temperature phase transitions and vapor pressure of less common ionic liquids based on the bis(trifuoromethanesulfonyl)imide anion.

Annalisa Paolone[1,*], Boumediene Haddad[2,3], Didier Villemin[3], Mostefa Boumediene[2], Bekhaled Fetouhi[4], Mohammed Amin Assenine[2,5]

[1] Consiglio Nazionale delle Ricerche, Istituto dei Sistemi Complessi, U.O.S. La Sapienza, Piazzale A. Moro 5, 00185 Roma, Italy.
[2] Chemistry Laboratory of Synthesis, Properties, and Applications (CLSPA-Saida), University of Saida, Algeria; haddadboumediene@yahoo.com, m.boumediene68@gmail.com
[3] LCMT, ENSICAEN, UMR 6507 CNRS, University of Caen, 6 bd Ml Juin, 14050 Caen, France; didier.villemin@ensicaen.fr
[4] Synthesis and Catalysis Laboratory LSCT, Tiaret University, Tiaret,Algeria; k.fetouhi@gmail.com
[5] Université Djillali Liabes, BP89, 22000 Sidi-Bel-Abbes, Algeria; asseninema@gmail.com
* Correspondence: annalisa.paolone@roma1.infn.it

Citation: Paolone, A.; Haddad, B.; Villemin, D.; Boumediene, M.; Fetouhi, B.; Assenine, M.A. Thermal Decomposition, Low Temperature Phase Transitions and Vapor Pressure of Less Common Ionic Liquids based on the Bis(trifuoromethanesulfonyl)imide Anion. *Materials* **2022**, *15*, 5255. https://doi.org/10.3390/ma15155255

Academic Editor: Andreas Taubert

Received: 14 July 2022
Accepted: 27 July 2022
Published: 29 July 2022

Publisher's Note: MDPI stays neutral with regard to jurisdictional claims in published maps and institutional affiliations.

Copyright: © 2022 by the authors. Licensee MDPI, Basel, Switzerland. This article is an open access article distributed under the terms and conditions of the Creative Commons Attribution (CC BY) license (https://creativecommons.org/licenses/by/4.0/).

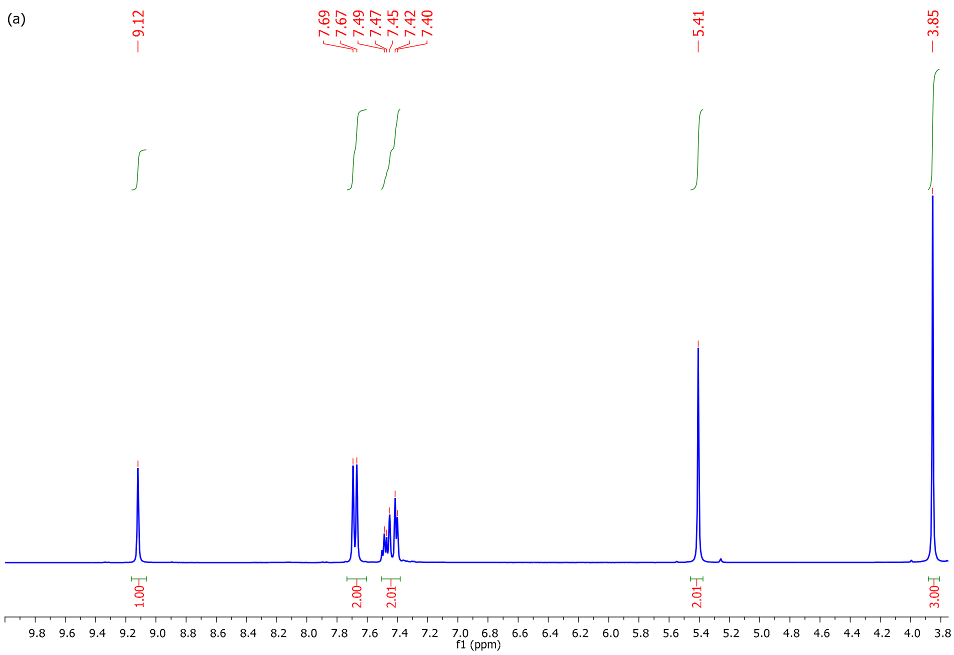

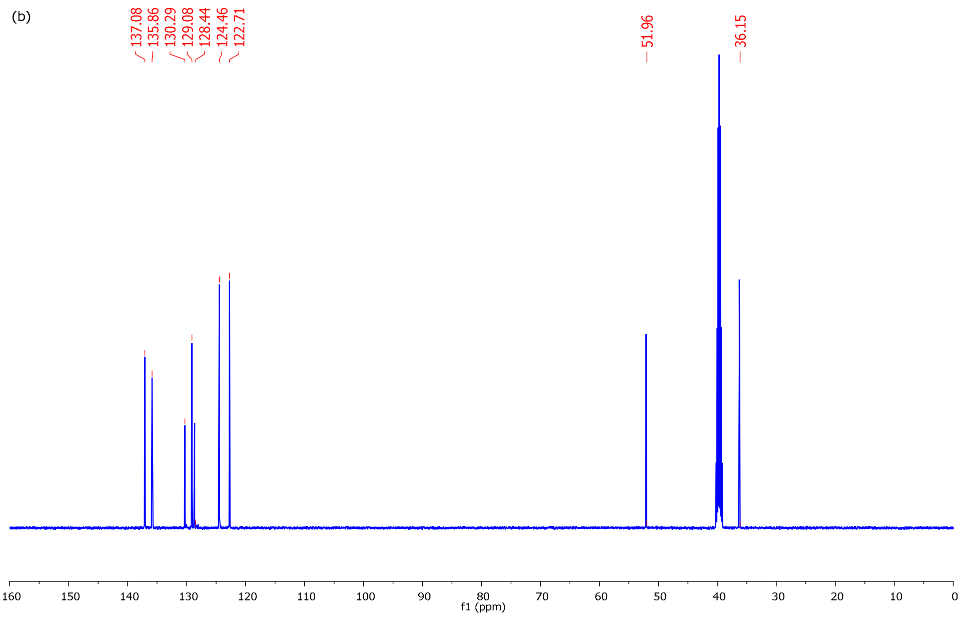

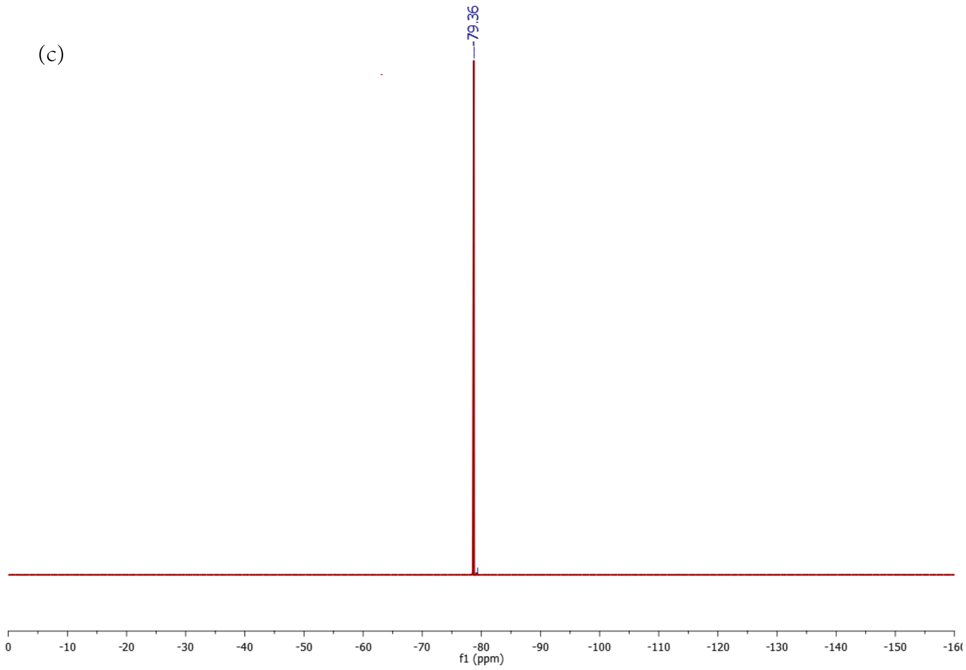

Figure S1. ^{1}H NMR (500 MHz) (**a**), ^{13}C NMR (125.75 MHz) (**b**) and ^{19}F NMR (470.62 MHz) (**c**) of [m- $C_6H_4(CH_2ImMe)^{2+}$][NTf_2^-]$_2$.

(a)

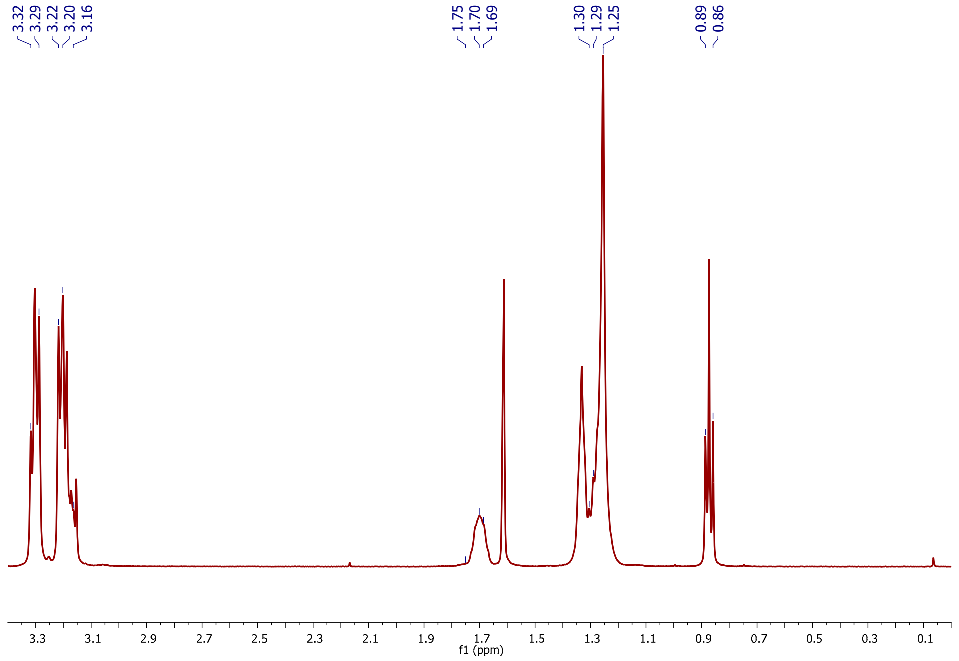
3.32
3.29
3.22
3.20
3.16
1.75
1.70
1.69
1.30
1.29
1.25
0.89
0.86
3.3 3.1 2.9 2.7 2.5 2.3 2.1 1.9 1.7 1.5 1.3 1.1 0.9 0.7 0.5 0.3 0.1
f1 (ppm)

(b)

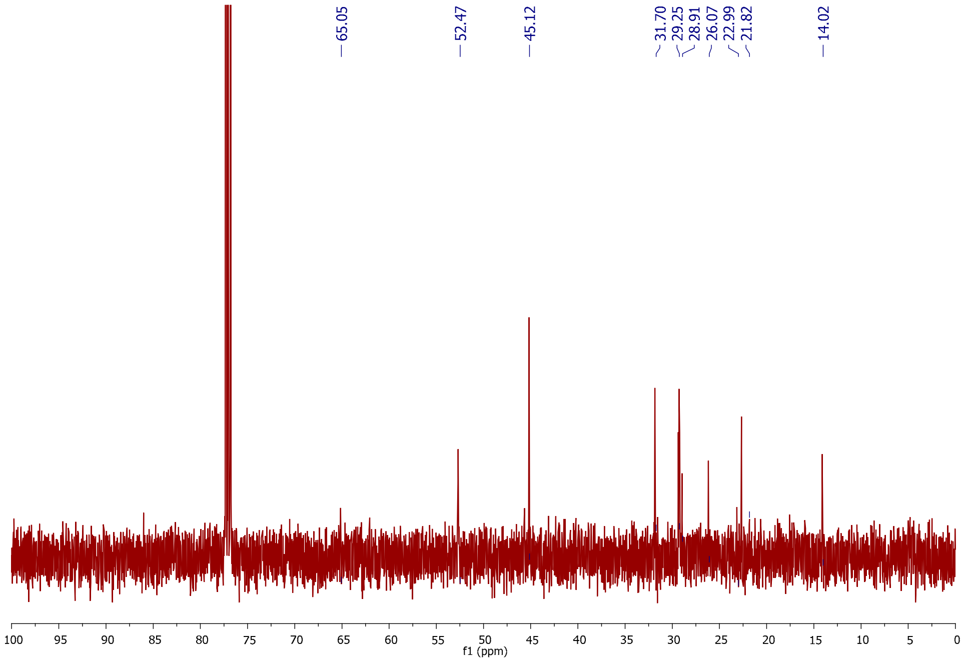
65.05
52.47
45.12
31.70
29.25
28.91
26.07
22.99
21.82
14.02
100 95 90 85 80 75 70 65 60 55 50 45 40 35 30 25 20 15 10 5 0
f1 (ppm)

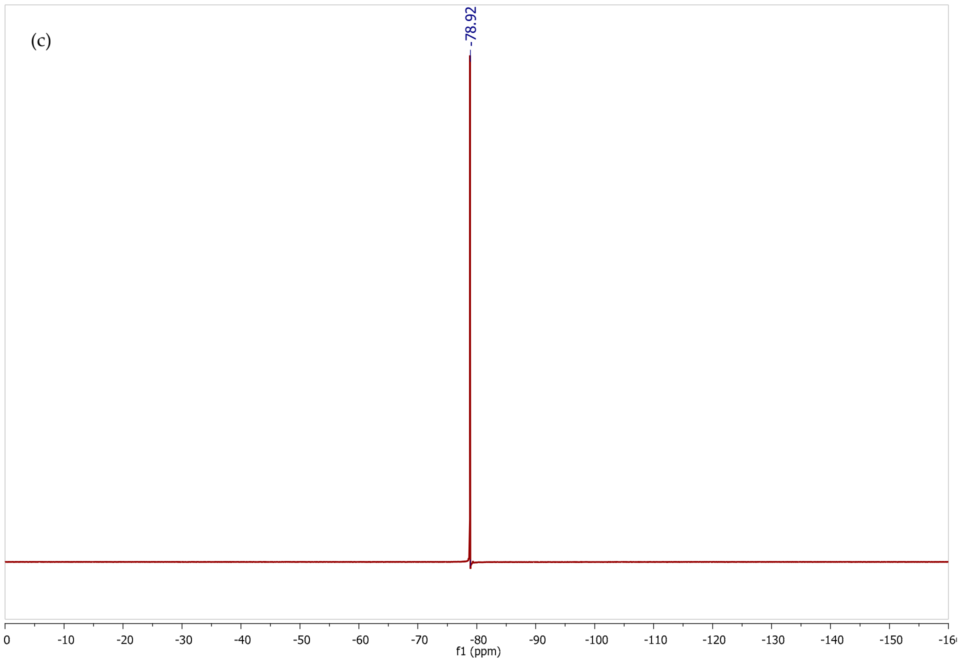

Figure S2. ^{1}H NMR (500 MHz) (**a**), ^{13}C NMR (125.75 MHz) (**b**) and ^{19}F NMR (470.62 MHz) (**c**) of [DABCO10$^+$][NTf$_2^-$].

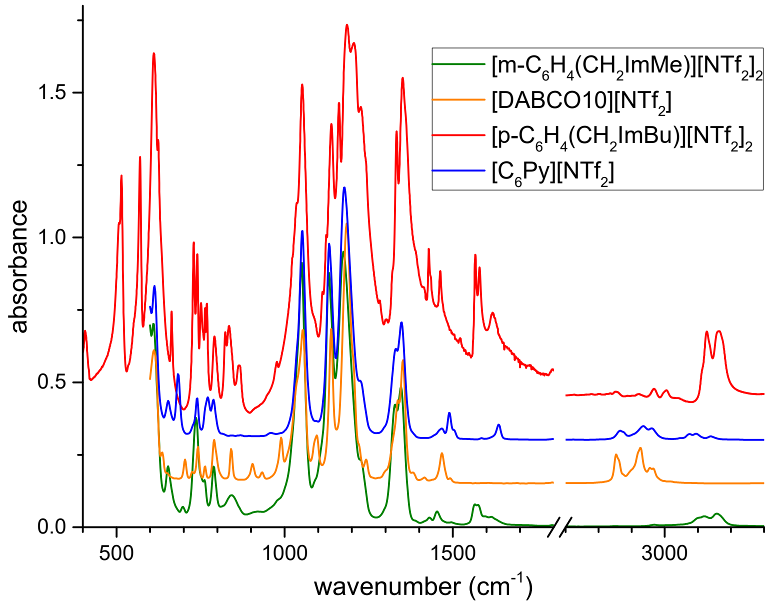

Figure S3. Infrared absorption spectra of the four ionic liquids.

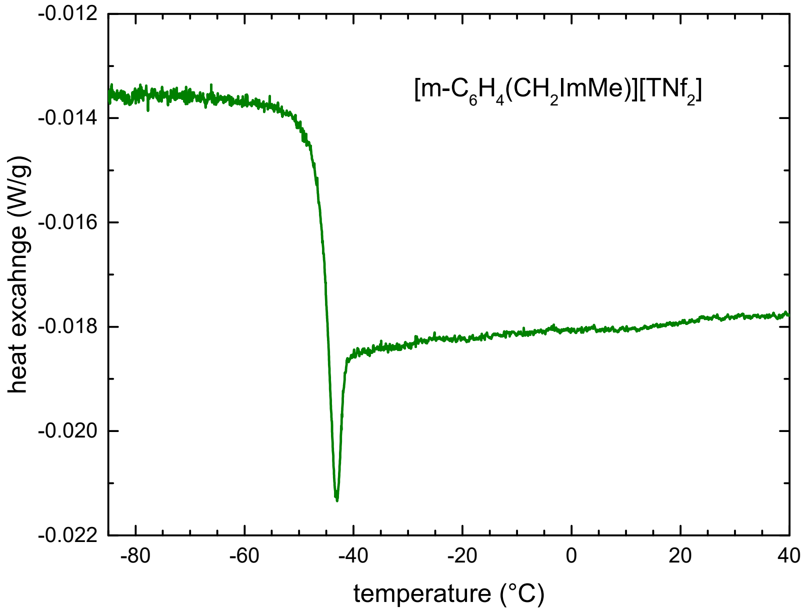

Figure S4. DSC trace of [m- $C_6H_4(CH_2ImMe)^{2+}$][NTf_2^-]$_2$, measured with a scanning rate of 1 °C/min.
